# A Monte Carlo study of IRTree models’ ability to recover item parameters

**DOI:** 10.3389/fpsyg.2023.1003756

**Published:** 2023-03-06

**Authors:** Gene M. Alarcon, Michael A. Lee, Dexter Johnson

**Affiliations:** ^1^Air Force Research Laboratory, Wright-Patterson Air Force Base, Dayton, Oh, United States; ^2^General Dynamics Information Technology, Inc., Atlanta, GA, United States

**Keywords:** item response theory, Monte Carlo, model fit, IRTree model, response style

## Abstract

Item response tree (IRTree) models are theorized to extract response styles from self-report data by utilizing multidimensional item response theory (IRT) models based on theoretical decision processes. Despite the growing popularity of the IRTree framework, there has been little research that has systematically examined the ability of its most popular models to recover item parameters across sample size and test length. This Monte Carlo simulation study explored the ability of IRTree models to recover item parameters based on data created from the midpoint primary process model. Results indicate the IRTree model can adequately recover item parameters early in the decision process model, specifically the midpoint node. However, as the model progresses through the decision hierarchy, item parameters have increased associated error variance. The authors ultimately recommend caution when employing the IRTree framework.

## Introduction

Item response theory (IRT) is a methodological framework for modeling response data that has gained support and interest over the past several decades. It encompasses a collection of mathematical models that can be applied to assess the relationship between test performance and the underlying trait (s) that drive that performance (Hambleton et al., 1991). The interest in IRT has led to development of an offshoot framework commonly known as item response trees (IRTrees). This methodology offers an alternative to the underlying assumptions of IRT by modeling theoretical decision hierarchies that underly response selection in surveys and tests (Böckenholt, 2012). Most commonly, these models have regularly been applied to examine response styles, as they are hypothesized to discretize specific response tendencies within both the data and model (e.g., Böckenholt, 2012; De Boeck and Partchev, 2012; Plieninger and Heck, 2018). Despite the growing popularity of IRTrees, there has been scant research that systemically examines the ability of IRTree models to recover item parameters of even the most regularly employed models in the IRTree framework across standard data parameters, such as sample size and test length. Researchers often compare IRTree models to traditional IRT models to determine fit, but only relative fit statistics are available for comparison, as IRTree models do not conform to absolute fit indices. As such, questions remain as to the ability of the models to recover the latent variables and item parameters from datasets. In this paper, we carried out a series of simulations to examine the influence of sample size and test length on one of the most widely employed IRTree models for assessing response style.

### Item response theory

IRT has many applications for studying test behavior (Baker, 2001; Embretson and Reise, 2013). In particular, the standard 2PL model has proven useful for analyzing dichotomous personality data (Waller and Reise, 1989; Reise and Waller, 1990). It is also a useful starting point for considering responses to Likert scales with more than two options. The equation for the 2PL model is:


(1) $$Pij\;\left(Y=1|\theta j\right)=\frac{\exp\left[\alpha_{i}\left(\theta_{j}-\beta_{i}\right)\right]}{1+\exp\left[\alpha_{i}\left(\theta_{j}-\beta_{i}\right)\right]}$$

where the probability of endorsing (Y = 1) item *i* for person *j* is a function of a person’s trait level (*θ _j_*), an item’s discrimination (*α*), and an item’s difficulty (*β*). In Equation 1, trait levels (*θ*) are assumed to have a mean of 0 and a standard deviation of 1, and item difficulty (*β_i_*) is placed onto the same metric as *θ*. In the personality context, the term item difficulty is used somewhat loosely. It reflects the location on the *θ* scale where individuals have a 50% probability of endorsing the item. More difficult items require higher *θ* for endorsement. In general, items are modeled to represent different degrees of the latent trait being measured. The change in probability levels out as individuals’ trait levels get farther away from the item’s difficulty. Based on this, the most difficult items can be useful for isolating individuals at top trait levels, whereas other items may be useful for partitioning individuals at a low-to-moderate standing on the trait.

Most self-report scales in the literature are not dichotomous, but rather comprised of polytomous items. Researchers have adapted the 2PL model to polytomous items, most notably with the graded response model (GRM; Samejima, 1969) and the graded partial credit model (GPCM; Masters, 1982). However, one of the key assumptions of IRT is that of local independence (Embretson and Reise, 2013). Local independence states that once the latent trait has been accounted for, the items are conditionally independent of each other. However, response styles may violate this assumption (Blalock, 1970). Participants who maintain a response style may respond in a fixed pattern, regardless of item content and their own corresponding latent trait (s) of interest, resulting in conditional dependency across items. As such, researchers have developed new multidimensional models to account for response styles that are derived from IRT methodology.

### IRTrees

**Figure 1:**
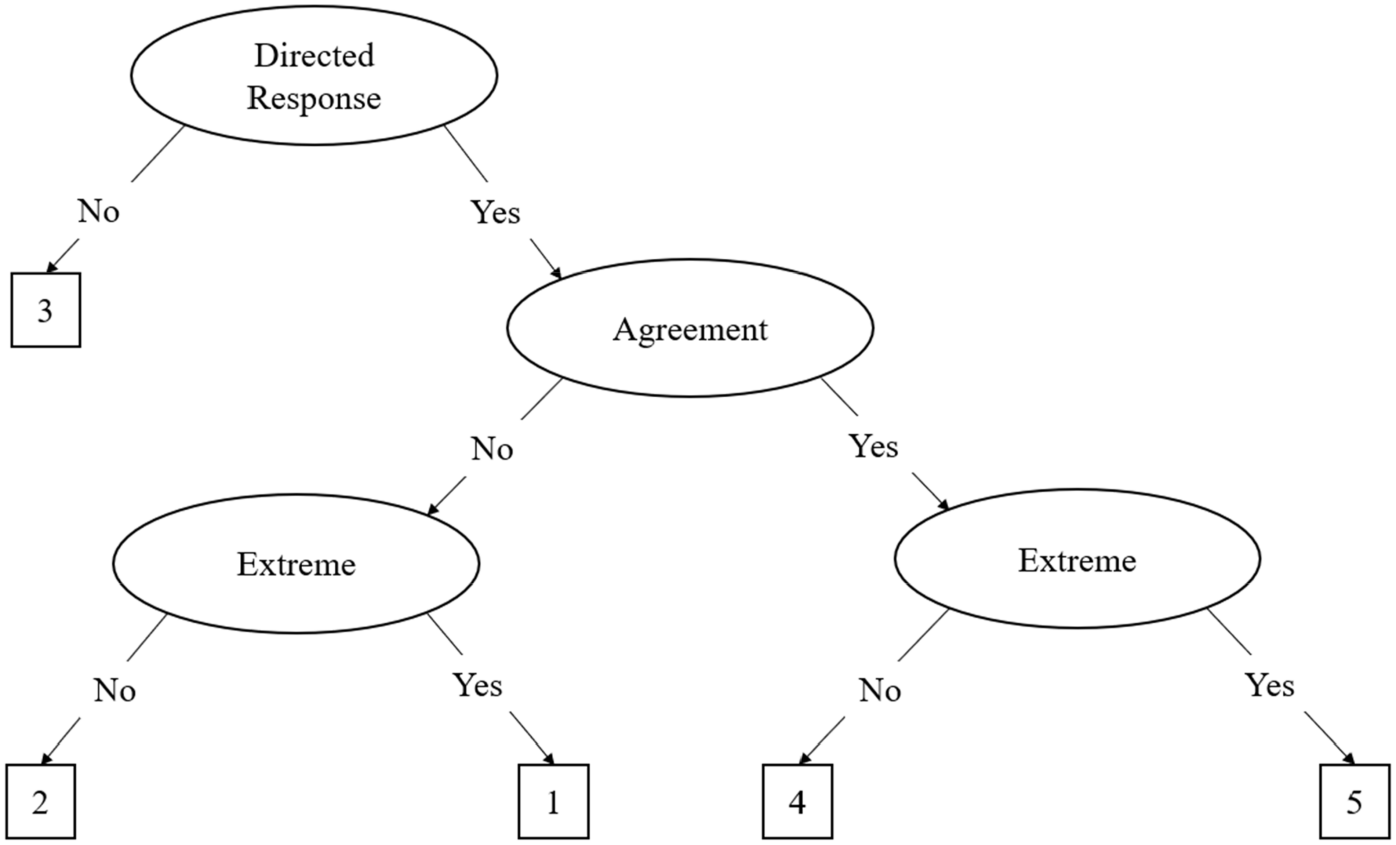
The Midpoint Primary Process (MPP) Decision Hierarchy Model. This figure assumes a “1” to “5” Likert scale for response options.

The IRTree framework was developed to better ascertain response styles in participant responding using non-compensatory multidimensional IRT models (Böckenholt, 2012; De Boeck and Partchev, 2012). The most common IRTree models can be differentiated into agreement primary process (APP) and midpoint primary process (MPP), where the former is primarily used for even-point scales and the latter is used for odd-point scales. For the rest of the paper, we focus on the 5-point MPP model, as 5-point Likert scales are the most common in the literature. The MPP model is based on a theoretical decision-making hierarchy where participants respond to items on a questionnaire using three decisions, called nodes, illustrated in Figure 1 and Table 1. The MPP model decomposes the original Likert data into three distinct nodes, comprised of binary decision processes. This decomposition shown in Figure 1 results in three distinct factors as represented in the following equation:


(2) $$\begin{array}{l} p\left(\gamma_{vi}\right)=\left[\frac{\exp\left(\alpha_{1vi}\left(\theta_{1j}-\beta_{1i}\right)\right)}{1+\exp\left(\alpha_{1vi}\left(\theta_{1j}-\beta_{1i}\right)\right)}\right]\left[\frac{\exp\left(\alpha_{2vi}\left(\theta_{2j}-\beta_{2i}\right)\right)}{1+\exp\left(\alpha_{2vi}\left(\theta_{2j}-\beta_{2i}\right)\right)}\right]\\ \left[\frac{\exp\left(\alpha_{3vi}\left(\theta_{3j}-\beta_{3i}\right)\right)}{1+\exp\left(\alpha_{3vi}\left(\theta_{3j}-\beta_{3i}\right)\right)}\right] \end{array}$$

where each node has its own *α* (discrimination) and *β* (difficulty) parameters which are multiplied to indicate endorsement of an individual item (γ_vi_). The first decision node indicates interindividual differences on the midpoint node, as represented by *θ*. Subsequent nodes indicate the interindividual differences on the agreement and extreme responding node, represented by *η*_1_ and *η*_2_, respectively. The discrimination and difficulty parameters represent said parameters for each node, respectively. As illustrated in Equation 2 and Figure 1, the decision hierarchy progresses through each node. Table 2 illustrates the transformation into pseudo-item matrices to represent this decision hierarchy.

**Table 1:** Category probabilities based on the MPP.

Response category	Probability formula
1	(1-θ(α_M_θ_M_ + β_M_))*(1-θ(α_A_θ_A_ + β_A_))*θ(α_E_θ_E_ + β_E_)
2	(1-θ(α_M_θ_M_ + β_M_))*(1-θ(α_A_θ_A_ + β_A_))*(1-θ(α_E_θ_E_ + β_E_))
3	θ(α_M_θ_M_ + β_M_))
4	(1-θ(α_M_θ_M_ + β_M_))*θ(α_A_θ_A_ + β_A_)*(1-θ(α_E_θ_E_ + β_E_))
5	(1-θ(α_M_θ_M_ + β_M_))*θ(α_A_θ_A_ + β_A_)*θ(α_E_θ_E_ + β_E_)

**Table 2:** Pseudo-items matrices for five-category model.

Category	Midpoint	Agreement	Extreme
1	0	0	1
2	0	0	0
3	1	–	–
4	0	1	0
5	0	1	1

Initially, participants must decide whether they have an opinion about the item, which is illustrated in the first node, known as the midpoint response (MRS) node. In this decision process, either the middle response option or a directed response is chosen. If a middle response is chosen the decision hierarchy is terminated, as the individual responds with the midpoint of the scale (e.g., a 3 on a 5-point Likert scale) and no further elaboration is required (see Figure 1). As illustrated in Table 1, only the MRS node influences a MRS of 3 on the scale. If a directed response is chosen, the participant continues down the decision hierarchy to the agreement node, choosing a directed response of agreement or disagreement (see agreement node in Figure 1). The last decision process in the hierarchy is the extreme node. In this process, the participant, having already chosen a directed response, must choose whether to respond in the extreme or not. This process is illustrated by the two extreme response (or ERS) nodes in Figure 1. Importantly, although the ERS node is represented by two nodes in the figure, there is only one latent trait for ERS as it is a function of the previous responses. As illustrated in Table 1, the ERS node has an influence on both the agreement and disagreement side of the scale, but it is one latent trait. In other words, the ERS node is a function of extreme or not extreme response, regardless of whether they previously decided to agree or disagree.

Researchers utilizing the IRTree methodology have advocated that it is more effective at managing the variance from response styles than traditional IRT models and other methods of response style identification (De Boeck and Partchev, 2012; Böckenholt, 2017). It is argued that the agreement node extracted from the multidimensional IRT model described above is a purer form of the latent construct because MRS and ERS have been controlled for in the model (De Boeck and Partchev, 2012; Böckenholt, 2017). Indeed, Lahuis et al. (2019) found the agreement node predicted job performance and other variables better than latent traits from the GRM model. However, other researchers have demonstrated the constructs of MRS and ERS have provided additional predictive validity in multiple contexts (e.g., He and Van de Vijver, 2015; Sun et al., 2019). In these cases, the nature of the data itself has not been known (as it is observed data rather than simulated data), which makes the effects of response style and the effects of other latent traits difficult to fully parse.

As IRTrees are relatively nascent, many questions about the models remain. Most studies conducted on IRTrees have been on observed data. Böckenholt’s seminal articles on IRTrees (De Boeck and Partchev, 2012; Debeer et al., 2017) utilized data from the Personal Need for Structure Scale (PNS; Neuberg and Newsom, 1993). Across articles he found the PNS fit the data better than traditional models, such as the GRM (Böckenholt, 2017; Böckenholt and Meiser, 2017). IRTrees have also been used to explore global scales such as the Five Factor Model (FFM) of personality (Khorramdel et al., 2019) and other more specific scales (e.g., subordinate traits of the FFM; LaHuis et al., 2019; Park and Wu, 2019). Previous research on IRTrees has traditionally focused on comparing IRTree models and unidimensional models of IRT (e.g., GRM and GPCM) with the Akaike information criterion (AIC; Akaike, 1974) and Bayesian information criterion (BIC, Schwarz, 1978). Meiser et al. (2019) have noted this as a limitation in the literature. Due to not being modeled on the same data, direct comparison between ordinal response data and IRT models with AIC and BIC violate a fundamental assumption of these statistics.

In addition, there have been some simulation studies in the literature on IRTrees. Some such studies have kept sample size and test length constant (Jin and Wang, 2014; Jeon and De Boeck, 2016; Plieninger and Heck, 2018), while others have either focused solely on one response style (e.g., ERS; Jin and Wang, 2014), scales with only three response options (Jeon and De Boeck, 2016), alternative response styles (e.g., acquiescence; Plieninger and Heck, 2018), or extending the general framework (Tijmstra et al., 2018; Ames and Myers, 2020). None have explored the ability of the model to recover parameters across sample sizes and test length using Monte Carlo simulation. Given the wide range of potential applications for IRTrees across numerous domains, understanding the ability of the IRTree models to recover item parameters and the necessary sample size and test length to do so are important questions for the framework.

### Current study

The current study sought to explore the ability of the IRTree model to recover parameters from data created from the IRTree methodology using the MPP model across sample sizes and test lengths. Specifically, we explored if the MPP model was able to recover item (discrimination and difficulty parameters) parameters from 5-point Likert data under varying test lengths and sample sizes. The current study used a 4 (sample size) x 3 (test length) design to examine these research questions.

## Method

### Simulation

#### Sample sizes

We chose to model data across fours sample sizes: 500, 1,000, 1,500, and 5,000. Wang and Nydick (2015) have suggested a minimum sample size of 1,000 are required for adequate parameter recovery of non-compensatory MIRT models. Notably, their study was conducted on data sets that did not have missing data. However, when items are recoded into the binary items for IRTree analysis, there is missing data based on the inherent structure of the hierarchical model (see Table 1). As some IRTree studies conducted in the literature are analyzed on data sets as small as 500 or less (e.g., Leventhal, 2019; Luby et al., 2020; Spratto et al., 2021), we chose the samples size of 500 to represent the common practice in the literature, 1,000 as a minimum threshold per Wang and Nydick (2015), and 1,500 as a moderately sized data set, and 5,000 as a large data set.

#### Test length

We chose test lengths of 10, 15, and 20 items. As noted by Lang et al. (2019), IRT models with less than 10 items are often unstable. Best practices set forth in the literature typically advocate for a scale of at least 10 items for adequate parameters. Additionally, given the number of latent traits that we intend to model, smaller scales may have difficulty converging with fewer items. After conversion of the data to the IRTree model, some of these items will be missing, due to the binary transformation. We chose an upper limit of 20 items because we deemed it unlikely to find Likert-type scales that are longer than 20 items per construct and do not violate local independence. Furthermore, previous simulation studies on IRTree models have used 20 item-scales in their stimulations (Jin and Wang, 2014; Jeon and De Boeck, 2016; Plieninger and Heck, 2018).

### Data creation and parameter recovery

To create the IRTree parameters, we created data based on the formula from Böckenholt (2017) illustrated in Equation 2 and Table 1.

#### Alpha parameters

We created three alpha matrices, one each for the midpoint, agreement, and extreme nodes for the discrimination parameters, sampling from three log-normal distributions. The first log-normal distribution was for the midpoint node, which had a mean of-0.5 and a standard deviation of 0.5, as established by previous research (Tijmstra et al., 2018). The agreement node was sampled from a log-normal distribution with a mean of 0.3 and a standard deviation 0.2, which is standard for a binary 2PL model (Mislevy and Stocking, 1989; Harwell and Baker, 1991; Mooney, 1997; Feinberg and Rubright, 2016; Bulut and Sünbül, 2017). Lastly, the discrimination parameters for the extreme node were sampled from a log-normal distribution with a mean of 0.5 and a standard deviation of 0.5. We chose this latter distribution because previous research has demonstrated the discrimination parameters for the extreme node are negatively associated with the midpoint node (Böckenholt and Meiser, 2017). As such, we took the inverse of the midpoint node. The mean *α* parameters for the 20-item scale were 0.49, 1.31, and 1.52 for the midpoint, agreement and extreme nodes, respectively.

#### Delta parameters

All difficulty parameters were drawn from three normal distributions. Previous models utilizing the IRTree methodology have modeled the difficulty parameters differently.^1^ Tijmstra et al. (2018) reasoned the midpoint node had a normal distribution with a mean of-2; whereas Ames and Myers (2020) argued the midpoint and extreme both had a mean of 0. Because there is little agreement on the mean of the midpoint node, we sampled parameters from a normal distribution with a mean of-1 and a standard deviation of 0.5 for the midpoint node, splitting the difference between the studies. Similarly, the extreme node tends to fall on the extreme side of the latent trait when difficulty parameters are modeled with IRTrees. As such, we randomly sampled difficulty parameters from a normal distribution with a mean of 1 and a standard deviation of 0.5. The difficulty parameters for the agreement node were drawn from a normal distribution with a mean of 0 and a standard deviation of 1, per previous research (Mislevy and Stocking, 1989; Harwell and Baker, 1991; Mooney, 1997; Feinberg and Rubright, 2016; Bulut and Sünbül, 2017). The mean *d* parameters for the 20-item scale were-0.44, −0.13, and 1.58 for the midpoint, agreement and extreme nodes, respectively.

#### Theta parameters

Ability parameters were drawn from a standard normal distribution. We specified the correlations between the latent traits based on previous research (Böckenholt, 2017). Specifically, the agreement node had a small average negative relationship with the midpoint node (*r* = −0.10) and a small average positive relationship with the extreme node (*r* = 0.10). The midpoint and extreme nodes were set to correlate with each other on average at −0.50, as previous research has demonstrated they have a modest negative correlation (Böckenholt, 2017).

#### Analysis

Data was modeled using functions from the following packages in R (version 4.1.3): *mirt* (Chalmers, 2012), *SimDesign* (Chalmers and Adkins, 2020), and *MASS* (Venables and Ripley, 2002). The IRTree data was created with the model specified in the above section, which resulted in binary items. A total of 12 conditions were evaluated with 2000 replicates each. We estimated all models using the Markov Chain Monte Carlo (MCMC) algorithm with the *R2Jags* (Su and Yajima, 2021) package in R. We chose this estimation as previous research has demonstrated the MCMC algorithm is the best for estimating non-compensatory models (Wang and Nydick, 2015). The *α* prior distributions were, respectively, set to-0.5, 0.3, and 0.5 for the midpoint, agreement, and extreme nodes, in accordance with our data creation. The delta prior distributions were set to-1, 0 and 1 for the midpoint, agreement, and extreme nodes respectively, in accordance with our data creation outlined above.

The accuracy of recovery for item parameters and theta were evaluated based on the root mean square error (RMSE) and the confidence intervals around the estimates. We chose the former statistic as they can illustrate the difference between the predicted scores and the actual scores the data was created on. We report the average standard errors and confidence intervals for the alpha and delta parameters for each node in each condition. To analyze the effects of the simulation conditions on parameter recovery we conducted a series of between-subjects factorial analysis of variance (ANOVA) to determine the effect each of the manipulations had on estimation bias. We examined the effect sizes of sample size and test length by regressing the manipulations as factors onto the RMSE of the discrimination and difficulty parameters. Partial eta-squared values were calculated using the *effectsize* package (Ben-Shachar et al., 2020).

## Results

Results of the recovery, mean parameters for each node, standard errors for each node and 95% confidence intervals for the discrimination and difficult parameters from the simulated IRTree data were obtained for the 12 conditions in the study. Table 3 illustrates the mean RMSE and bias statistics for the discrimination and difficulty parameters across sample size and test length. Table 4 illustrates the mean parameter estimate, standard error and 95% confidence interval for the alpha and delta parameters across sample size and test length.

**Table 3:** Parameter recovery statistics for the IRTree MPP model fitted to IRTree MPP model data.

Model node	*N*	Test length	*a* RMSE	*a* Bias	*d* RMSE	*d* Bias
Midpoint	10	500	0.13	0.00	0.06	−0.02
		1,000	0.11	−0.02	0.09	0.03
		1,500	0.11	−0.01	0.08	−0.02
		5,000	0.16	−0.06	0.09	−0.01
	15	500	0.15	0.03	0.11	0.01
		1,000	0.14	0.01	0.10	0.00
		1,500	0.09	0.02	0.11	0.05
		5,000	0.15	−0.06	0.10	0.04
	20	500	0.17	0.01	0.07	−0.04
		1,000	0.16	−0.08	0.10	−0.03
		1,500	0.14	−0.05	0.11	−0.02
		5,000	0.10	−0.01	0.07	−0.03
Agreement	10	500	0.45	−0.29	0.91	−0.91
		1,000	0.31	−0.31	0.96	−0.96
		1,500	0.40	−0.40	0.86	−0.86
		5,000	0.49	−0.49	0.81	−0.81
	15	500	0.54	−0.46	0.94	−0.94
		1,000	0.46	−0.43	0.88	−0.88
		1,500	0.44	−0.43	0.96	−0.96
		5,000	0.37	−0.35	1.04	−1.04
	20	500	0.53	−0.46	0.92	−0.92
		1,000	0.41	−0.41	0.95	−0.95
		1,500	0.38	−0.37	0.93	−0.93
		5,000	0.35	−0.34	0.97	−0.97
Extreme	10	500	1.03	−1.03	2.91	−2.91
		1,000	1.15	−1.15	2.93	−2.93
		1,500	0.99	−0.99	2.87	−2.87
		5,000	0.90	−0.90	2.94	−2.94
	15	500	0.59	−0.58	3.12	−3.12
		1,000	0.81	−0.80	3.01	−3.01
		1,500	0.88	−0.88	3.00	−3.00
		5,000	0.73	−0.71	2.94	−2.94
	20	500	0.98	−0.98	3.33	−3.33
		1,000	1.10	−1.10	3.34	−3.34
		1,500	1.13	−1.12	3.30	−3.30
		5,000	0.99	−0.99	3.32	−3.32

**Table 4:** Parameter recovery statistics for the IRTree MPP model fitted to IRTree MPP model data.

Model node	*N*	Test length	*a X̅*	*a* SE	*a* 95% CI	*d X̅*	*d* SE	*d* 95% CI
Midpoint	10	500	0.52	0.01	0.50, 0.54	−0.44	0.01	−0.46, −0.42
		1,000	0.50	0.01	0.48, 0.52	−0.39	0.01	−0.41, −0.37
		1,500	0.51	0.01	0.49, 0.52	−0.44	0.01	−0.46, −0.37
		5,000	0.46	0.01	0.44, 0.48	−0.43	0.01	−0.45, −0.41
	15	500	0.56	0.01	0.54, 0.58	−0.43	0.01	−0.45, −0.41
		1,000	0.54	0.01	0.52, 0.56	−0.44	0.00	−0.44, −0.44
		1,500	0.55	0.01	0.53, 0.57	−0.39	0.01	−0.41, −0.37
		5,000	0.47	0.01	0.45, 0.49	−0.40	0.00	−0.40, −0.40
	20	500	−0.48	0.01	−0.50, −0.46	−0.48	0.01	−0.50, −0.46
		1,000	−0.47	0.01	−0.49, −0.45	−0.47	0.01	−0.49, −0.45
		1,500	−0.45	0.01	−0.47, −0.43	−0.45	0.01	−0.47, −0.43
		5,000	−0.47	0.01	−0.49, −0.45	−0.47	0.01	−0.49, −0.45
Agreement	10	500	0.98	0.01	0.96, 1.00	−1.11	0.01	−1.13, −1.09
		1,000	0.96	0.01	0.94, 0.98	−1.17	0.01	−1.19, −1.15
		1,500	0.87	0.01	0.85, 0.89	−1.06	0.01	−1.08, −1.04
		5,000	0.78	0.01	0.76, 0.80	−1.01	0.01	−1.03, −0.99
	15	500	0.82	0.01	0.80, 0.84	−1.15	0.01	−1.17, −1.13
		1,000	0.85	0.01	0.83, 0.87	−1.08	0.01	−1.10, −1.06
		1,500	0.85	0.01	0.83, 0.87	−1.16	0.01	−1.18, −1.14
		5,000	0.93	0.01	0.91, 0.95	−1.24	0.01	−1.26, −1.22
	20	500	−1.05	0.01	−1.07, −1.03	−1.05	0.01	−1.07, −1.03
		1,000	−1.08	0.01	−1.10, −1.06	−1.08	0.01	−1.10, −1.06
		1,500	−1.06	0.01	−1.08, −1.04	−1.06	0.01	−1.08, −1.04
		5,000	−1.10	0.01	−1.12, −1.08	−1.10	0.01	−1.12, −1.08
Extreme	10	500	0.30	0.02	0.26, 0.34	−1.71	0.01	−1.73, −1.69
		1,000	0.17	0.03	0.11, 0.23	−1.73	0.01	−1.75, −1.71
		1,500	0.34	0.02	0.30, 0.38	−1.67	0.01	−1.69, −1.65
		5,000	0.43	0.02	0.39, 0.47	−1.74	0.01	−1.76, −1.72
	15	500	0.67	0.02	0.63, 0.71	−1.90	0.01	−1.92, −1.88
		1,000	0.45	0.02	0.41, 0.49	−1.78	0.01	−1.80, −1.76
		1,500	0.36	0.02	0.32, 0.40	−1.77	0.01	−1.79, −1.75
		5,000	0.54	0.02	0.50, 0.58	−1.71	0.01	−1.73, −1.69
	20	500	−1.75	0.01	−1.77, −1.73	−1.75	0.01	−1.77, −1.73
		1,000	−1.76	0.01	−1.78, −1.74	−1.76	0.01	−1.78, −1.74
		1,500	−1.72	0.01	−1.74, −1.70	−1.72	0.01	−1.74, −1.70
		5,000	−1.74	0.01	−1.76, −1.72	−1.74	0.01	−1.76, −1.72

### Item parameter recovery

#### Discrimination parameters

**Figure 2:**
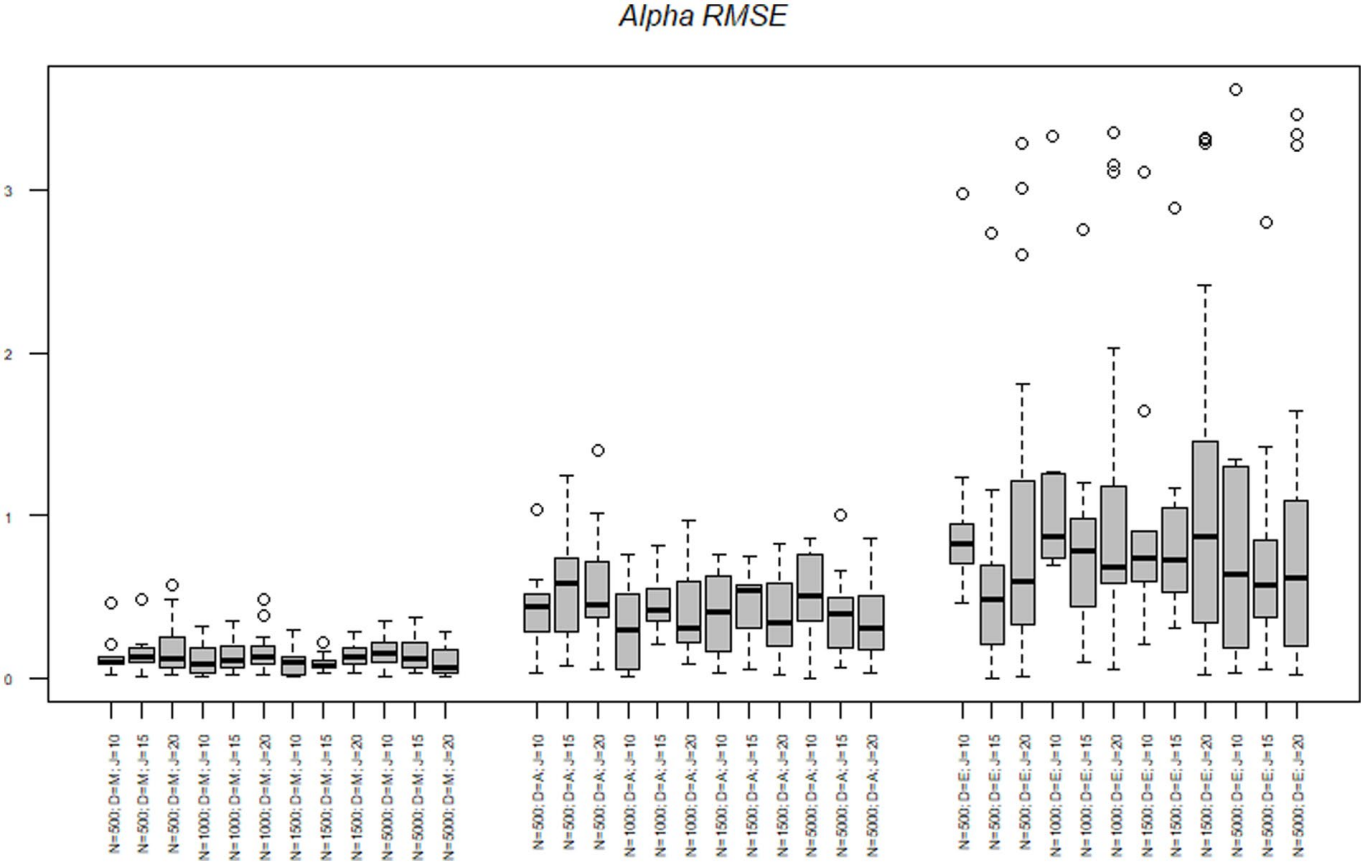
Alpha parameter RMSE across nodes, sample size, and test length. *N* = Sample size; *D* = Model node; *J* = Number of items.

First, we discuss the alpha parameters of each node. The RMSE of the midpoint node ranged from 0.00 to 0.56 with a mean of 0.13. The RMSE of the agreement node ranged from 0.00 to 1.39 with a mean of 0.42. The RMSE of the extreme node ranged from 0.03 to 3.61 with a mean of 0.94. Results of the alpha parameter RMSE across conditions are illustrated in Figure 2. Results indicate that as the model progressed through the decision hierarchy it had less ability to recover the alpha parameters of the model, as there was more variance in the estimate, with the most dramatic change occurring between the agreement and extreme nodes. As illustrated in Figure 2, the midpoint node had the lowest RMSE followed by the agreement node, followed then by the extreme node which was more than triple the RMSE of the agreement node. Figure 2 also illustrates the upper and lower quartiles for the Midpoint node are relatively small, but as the model increases through the hierarchy the quartiles and extremes (upper and lower) become larger and the outliers become more pronounced. Table 4 illustrates the mean alpha parameters, as well as the associated standard error and confidence intervals. There are several issues to note. First, the standard errors and confidence intervals of all the alpha parameters are relatively small, with the standard error ranging from 0.01 to 0.03. As noted before, the mean alpha parameters were 0.49, 1.31, and 1.52, for the midpoint, agreement, and extreme nodes, respectively. The midpoint and agreement nodes were close to their respective true means. The 10-, 15-, and 20-item test lengths were all close to their mean distributions.

To better understand the effects of the sample size, test length and nodes on the RMSE we conducted a series of analysis of variance (ANOVAs) to determine the amount of variance each factor accounted for in the criterion. The effects of sample size, test length, and node on the alpha parameters are illustrated in Table 5. Across all criteria the node accounted for the most variance in the RMSE statistics. The node accounted for 27% of the variance in the alpha RMSE and 2% of the variance was due to an interaction between the node and test length. Simple effects illustrated the midpoint node had the lowest RMSE and was significantly different from the agreement *t*(504) = −4.96, *p* < 0.001 and the extreme node *t*(504) = −13.61, *p* < 0.001. Additionally, the agreement and extreme nodes were significantly different from each other *t*(504) = −8.64, *p* < 0.001, with the agreement node having a smaller average RMSE.

**Table 5:** Accounted variance for parameter recovery metrics for the IRTree MPP model fitted to IRTree MPP model data.

Model node	Variable	*a* RMSE	*d* RMSE
Sample size		0.00	0.00
Test length		0.00	**0.01**
Node		**0.27**	**0.81**
Sample size * Test length		0.00	0.00
Sample size* Node		**0.02**	**0.02**
Sample size * Test length * Node		0.00	0.00

*N* = 450. Values are partial eta-squared. Significant values (*p* < 0.05) are in bold.

Next, we explored the interaction effect to understand the interaction of node and test length. For clarity’s sake, we only refer to differences between nodes within tests. First, in the 10-item scale we found a significant difference in RMSE between the midpoint node and extreme *t*(504 = −7.36, *p* < 0.001 nodes and the agreement and extreme nodes *t*(504) = −4.99, *p* < 0.001. No differences were found between the midpoint and agreement nodes *t*(504) = 2.37, *p* = 0.30. In the 15 item scale there were no significant differences in RMSE between the agreement and extreme nodes *t*(504) = −3.06, *p* = 0.06. However, there was a significant difference between the midpoint and agreement nodes *t*(504) = −3.24, *p* = 0.03 and the midpoint and extreme nodes *t*(504) = −6.65, *p* < 0.001. Lastly, in the 20-item scale, all three nodes were significantly different from each other [midpoint-agreement *t*(504) = −3.24, *p* = 0.03; midpoint-extreme *t*(504) = −10.63, *p* < 0.001; and agreement-extreme *t*(504) = −7.39, *p* < 0.001]. In summation, as the test length became longer the difference in the discrimination RMSE between the nodes became larger.

#### Delta parameters

**Figure 3:**
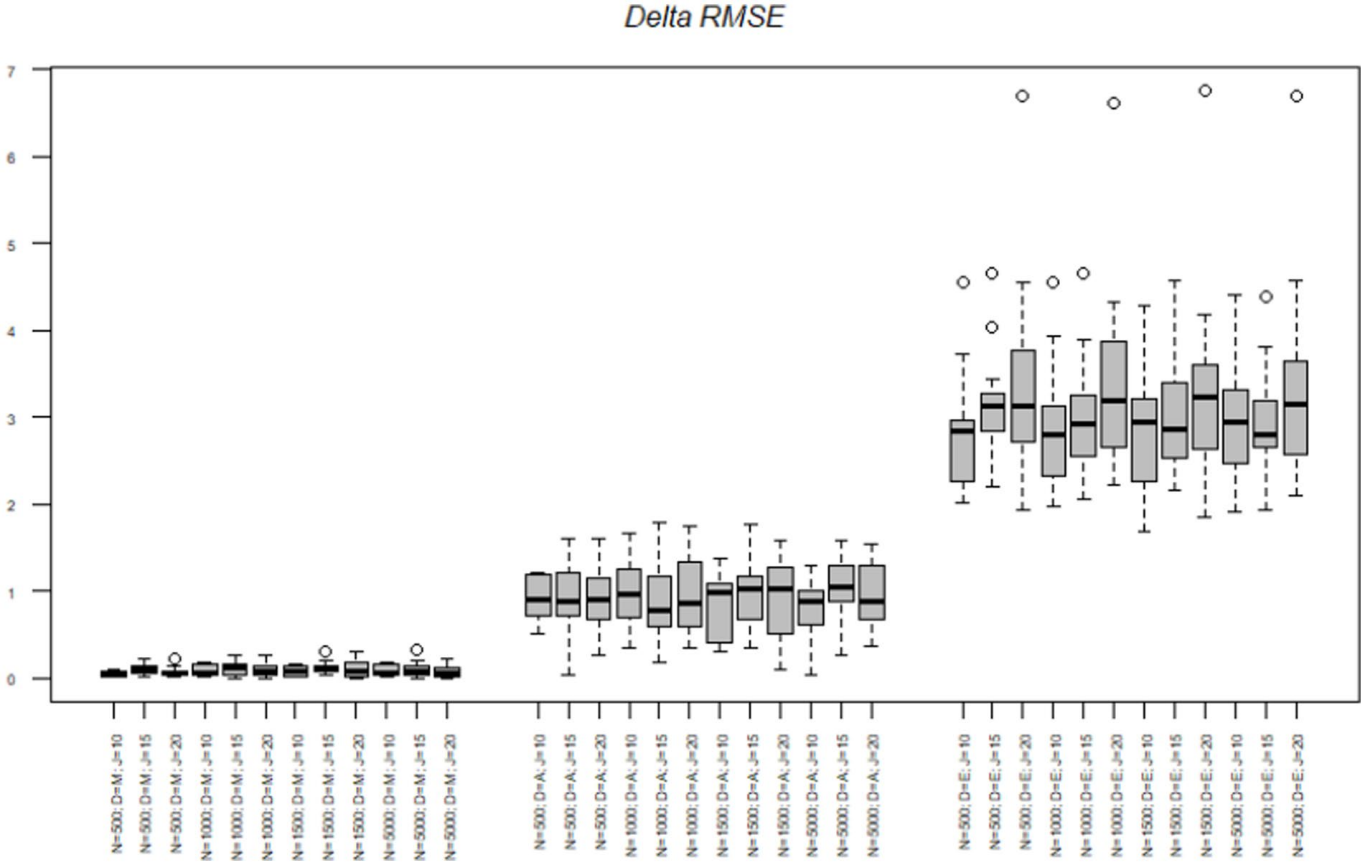
Delta parameter RMSE across nodes, sample size, and test length. *N* = Sample size; *D* = Model node; *J* = Number of items.

Next, we tested the delta parameters. The RMSE of the midpoint node ranged from 0.00 to 0.31 with a mean of 0.09. The RMSE of the agreement node ranged from 0.03 to 1.78 with a mean of 0.93. The RMSE of the extreme node ranged from 1.69 to 6.75 with a mean of 3.13. Figure 3 illustrates the same pattern as the alpha RMSE occurs with the delta RMSE, such that the midpoint node had the lowest delta RMSE, followed by the agreement node and then the extreme node which again had over triple the RMSE value of the other two nodes. Table 4 illustrates the mean delta parameter, the standard error associated with the estimate and the confidence intervals of the estimate. As noted, before, the true mean of the midpoint, agreement and extreme nodes were-0.43, −0.12, and 1.58, respectively. The average midpoint node was close to the true estimates. However, the agreement node was off by almost 10 times the true amount with an average of-1.10 whereas the true estimate was-0.12. Similarly, the extreme node was in the opposite direction with a mean delta of-1.74 and the true average delta being 1.58. Again, as with the alpha node, the standard errors were relatively small ranging from 0.00 to 0.01.

To better understand the effects of the sample size, test length and nodes on the difficulty RMSE we conducted a series of analysis of variance (ANOVAs) to determine the amount of variance each factor accounted for in the criterion. The effects of sample size, test length, and node on the alpha parameters are illustrated in Table 5. Across all criteria the node accounted for the most variance in the RMSE statistics. The node accounted for 81% of the variance in the delta RMSE, test length accounted for 1% of the variance and 2% of the variance was due to an interaction between the node and test length. Simple effects illustrated the midpoint node had the lowest RMSE and was significantly different from the agreement *t*(504) = −13.99, *p* < 0.001 and the extreme node *t*(504) = −50.05, *p* < 0.001. Additionally, the agreement and extreme nodes were significantly different from each other *t*(504) = −30.06, *p* < 0.001.

Next, we explored the interaction effect to understand the interaction of node and test length on the delta RMSE. For clarity’s sake, we only refer to differences between nodes within tests. First, in the 10-item scale we found a significant difference in RMSE between all the nodes [midpoint-agreement *t*(504 = −6.61, *p* < 0.001; midpoint-extreme nodes *t*(504) = −23.25, *p* < 0.001; and agreement-extreme nodes *t*(504) = −16.63, *p* < 0.001]. For the 15-items scale we found a significant difference between all the node [midpoint-agreement *t*(504 = −8.53, *p* < 0.001; midpoint-extreme nodes *t*(504) = −29.26, *p* < 0.001; and agreement-extreme nodes *t*(504) = −20.73, *p* < 0.001]. Lastly, the 20-item scale had significant differences between all nodes [midpoint-agreement *t*(504 = −9.93, *p* < 0.001; midpoint-extreme nodes t(504) = −37.53, *p* < 0.001; and agreement-extreme nodes *t*(504) = −27.59, *p* < 0.001]. As with the alpha parameter, the delta RMSE differences between the nodes increased as the test length increased.

## Discussion

The proper measurement and identification of latent variables is important for any psychometric methodology. With the growing popularity of IRTrees, understanding the influences of test length and sample size on the recovery of item parameters with the model is important. As we rarely know the actual item parameters of a population, it is important to also understand the ability of MPP models to recover their parameters and the impact of model misspecification on the IRTree framework, especially given its frequent comparison to traditional IRT models. The present study provided an evaluation of the IRTree framework using simulated data, exploring the ability of one of its most popularized models to recover item parameters, across sample sizes and test lengths.

### IRTree parameter recovery

First, we note the current paper is the first of its kind to manipulate both test length and sample size to determine their effect on the item and person parameter recovery in IRTree models. Previous research has constrained their test lengths to 20 or more items (Jin and Wang, 2014; Wang and Nydick, 2015; Jeon and De Boeck, 2016; Plieninger and Heck, 2018). In this study, test length accounted for an average of 1% of the error variance in recovering delta parameters. Interestingly, this difference occurred in the longer scales. This may be due to how MCMC estimates data patterns. The MCMC method uses a chain to estimate the parameters in a model (Kruschke, 2014). However, the missing data inherent in an IRTree model may lead the chain astray as it attempts to estimate the model parameters. This may lead to the wildly different parameters down the decision hierarchy seen in the current study. Interestingly, in the current study it was not sample size or test length that had the strongest impact on RMSE values but instead the node in the decision model.

Across the recovery of item parameters, we noticed an interesting trend. As the MPP model progressed through the decision hierarchy, the model was increasingly less accurate. The model was able to recover the discrimination and difficulty parameters for the midpoint with little error variance between the parameter estimates and the true parameters. However, as the model progressed, error variance increased exponentially. Ames and Myers (2020) note the missing values in the agreement and extreme nodes contribute to the increased error as the decision hierarchy progresses, but they found this increase to be negligible. However, there are a few key distinctions between our study and theirs. First, the primary focus of their paper was extending the IRTree framework to include person covariates, rather than testing the overall framework. As such, they did not explore the item parameters. The current study demonstrates there is substantial error in item discrimination and difficulty as the decision hierarchy progresses, which can become problematic. Second, they chose a mean of zero for their extreme and midpoint nodes and sampled their difficulty parameters from a normal distribution of 0. The difference in distributions means of the item parameters may have influenced the models in the current study. However, as they noted, little research has explored the parameters of the midpoint and extreme nodes. Accordingly, there remains conflicting information about their underlying parameters. Given this dearth of consensus, we chose a mean of the distribution between that provided by Tijmstra et al. (2018) and by Ames and Myers (2020), settling on-1 and 1 for the midpoint and extreme nodes, respectively. Further research is needed on the effect of the distribution of the parameters the ability of the model to recover the item parameters. Additionally, it would be fruitful for researchers to publish their discrimination and difficulty parameters in the literature, to assist researchers in understanding the effects of distributions across scale types.

The inclusion of a decision hierarchy appears to add additional error variance to the model when the response style variables are actually present. This is problematic, as previous researchers have used the extreme node to predict criterion or otherwise explain additional model variance (e.g., He and Van de Vijver, 2015; LaHuis et al., 2019). Additionally, not all people may respond with a response style. As such, mixture models where some participants can be classified as using response styles and other participants responding without response styles may be necessary (see Tijmstra et al., 2018) It may be the additional variance accounted for by the extreme node accounts is due to error in the model, rather than actual variance in observed scores. We advise that researchers should be cautious when utilizing variables from later in the decision hierarchy, such as the extreme node.

Despite the inability of the IRTree model to recover parameters of the model throughout the hierarchy, it may be beneficial to continue to research IRTrees. As we noted above, there is still debate on the distributions of the alpha and delta parameters. It may be that the parameters reported in the literature and explored in the current study are not the actual parameters of midpoint and extreme responding nodes. If so, this could have led to the model hierarchy issues described above. We have noted that as the decision hierarchy progresses, the model becomes increasingly less able to recover parameters. However, the current study only used a five-point Likert scale for the current analyzes, as we were more interested in sample size and test length’s effects on the model. It remains to be seen if different lengths of Likert-type scale responding determine influence the decision hierarchy. Additionally, the current study did not model the APP, which the IRTree framework was originally built on. The IRTree framework may be more amendable to the APP model rather than the MPP.

### Recommendations

We have several recommendations for IRTrees based on the current study. First, the IRTree framework, as modeled with the MPP model, can readily recover the item and person parameters from the earlier part of the decision model (i.e., the midpoint node). However, researchers should be cautious about interpreting parameters that are further down the decision hierarchy. In the current study, once the third decision node was reached, there was a large difference between the estimated parameter and actual parameter. This is not as problematic when the construct of interest is the second node (i.e., the latent trait), as in most models. However, as the model progresses, it becomes increasingly unstable, possibly due to the missing data.

Second, the general utility of the IRTree framework must be viewed cautiously. The IRTree framework increases error as the model progresses through the decision hierarchy, possibly due to the missing data when the decision hierarchy progresses past the midpoint node. For example, a common criticism of decision trees, which IRTrees are based on, is that small changes in the data can lead to large changes in the structure of the optimal decision tree (Kamiński et al., 2018). We only tested a five-point MPP model in the current study, so it remains to be seen how much error occurs in scales with more or fewer Likert responses. For example, Böckenholt (2017) explored a six-point Likert scale with IRTress with midpoint, weak agreement, strong agreement, and extreme nodes. The validity of the nodes further down the decision hierarchy may be suspect, given the amount of additional error added at each step. In this model, the separation of agreement into weak and strong nodes may lead to more error in the strong agreement node, as it is further down the decision hierarchy.

### Limitations and future research

The current study is not without limitations. First, we note the issue of the distributions of the response style parameters. We chose a distribution with means of-1 and 1 for the midpoint and extreme nodes, as there is little research on the distribution of these nodes. This may have impacted the results of the analyzes conducted on the data created on the MPP model. We have referenced previous research on the subject, but future research should continue to investigate and refine the most psychometrically appropriate mean distribution on these nodes, as well as alternative IRTree models. We assumed the extreme distribution was similar to the midpoint given previous research, but this may not be the case. Second, the current study focused on five-point Likert scales but did not explore the impact of IRTrees on other survey formats. For example, Böckenholt (2017) and Plieninger et al. (2018) have explored IRTrees and response styles across Likert, funnel, and drag-and-drop response scales. IRTrees may not have issues of compounding error in the other formats, as they do with the Likert scale format. Similarly, this study limited its scope to the commonly employed MPP model on a five-point scale, but there are other existing applications of the IRTree framework that vary in both overall model arrangement and the types of scales to which they are applied. Models for binary-coded data (i.e., testing data coded for accuracy; Debeer et al., 2017), as well as a range of polytomous scales (Jeon and De Boeck, 2016; Dibek, 2019; Ames, 2021; Spratto et al., 2021) have been developed, some of which are structurally distinct from the MPP decision hierarchy (e.g., Forthmann et al., 2019). Even the MPP model has been adapted to scales with varying response option lengths, altering the number steps in the decision hierarchy to accommodate for the number of response choices available (Plieninger and Meiser, 2014). Given the issues with IRTree model fit raised here naturally arise from the interaction of the model’s structure with the format of the scale, additional investigation should be conducted on how these elements could impact any given model’s ability to recover item parameters and recreate data.

## Conclusion

Though the growing interest and investigation surrounding IRTrees continues to show promise, it is important to consider the potential limitations and costs of the methodology. While this study found that IRTree models can sufficiently recover item parameters and recreate data to an extent, the IRTree model structure has the inherent drawback of inflating error variance due to missing data following pseudo-item transformation, which is compounded further as missing data accumulates over the course of the decision hierarchy. We urge researchers to take these limitations into consideration when employing IRTrees. While the framework has already demonstrated an exciting versatility in its capacity for modeling response styles, judicious application is essential for it to find its place within the repertoire of psychometricians across all fields.

## Data availability statement

The original contributions presented in the study are available from the corresponding author upon request.

## Author contributions

GA designed the study, analyzed the results and wrote the manuscript. ML analyzed the results and wrote the manuscript. DJ created all RJags models, analyzed the results and wrote the manuscript. All authors contributed to the article and approved the submitted version.

## Conflict of interest

ML was employed by General Dynamics Information Technology, Inc.

The remaining author declares that the research was conducted in the absence of any commercial or financial relationships that could be construed as a potential conflict of interest.

## Publisher’s note

All claims expressed in this article are solely those of the authors and do not necessarily represent those of their affiliated organizations, or those of the publisher, the editors and the reviewers. Any product that may be evaluated in this article, or claim that may be made by its manufacturer, is not guaranteed or endorsed by the publisher.

## Footnotes

^1^We note that we utilized delta parameters instead of beta parameters for the current study. Beta parameters are modified by alpha. As such, we wanted to test the unmodified difficulty parameters.
